# Nucleoporin 85 interacts with influenza A virus PB1 and PB2 to promote its replication by facilitating nuclear import of ribonucleoprotein

**DOI:** 10.3389/fmicb.2022.895779

**Published:** 2022-08-16

**Authors:** Yue-Huan Ling, Hao Wang, Mei-Qing Han, Di Wang, Yi-Xiang Hu, Kun Zhou, Yan Li

**Affiliations:** ^1^Department of Veterinary Medicine and Institute of Preventive Veterinary Sciences, Zhejiang University College of Animal Sciences, Hangzhou, Zhejiang, China; ^2^Hainan Institute, Zhejiang University, Sanya, Hainan, China; ^3^Zhejiang Provincial Key Laboratory of Preventive Veterinary Medicine, Hangzhou, Zhejiang, China

**Keywords:** influenza A virus, NUP85, RNP, PB1, PB2, nuclear import

## Abstract

Transcription and replication of the influenza A virus (IAV) genome take place in the nucleus of infected cells, which rely on host factors to aid viral ribonucleoprotein (vRNP) to cross the nuclear pore complex (NPC) and complete the bidirectional nucleocytoplasmic trafficking. Here, we showed that nucleoporin 85 (NUP85), a component of NPC, interacted with RNP subunits polymerase basic 1 (PB1) and polymerase basic 2 (PB2) in an RNA-dependent manner during IAV infection. Knockdown of NUP85 delayed the nuclear import of vRNP, PB1 and PB2, inhibiting polymerase activity and ultimately suppressing viral replication. Further analysis revealed that NUP85 assisted the binding of PB1 to nuclear transport factor Ran-binding protein 5 (RanBP5) and the binding of PB2 to nuclear transport factor importin α1 and importin α7. We also found that NUP85 expression was downregulated upon IAV infection. Together, our study demonstrated that NUP85 positively regulated IAV infection by interacting with viral PB1 and PB2, which may provide new insight into the process of vRNP nuclear import and a novel target for effective antivirals.

## Introduction

Influenza A virus (IAV) belongs to the family *Orthomyxoviridae* and represents important pathogens of humans and animals. IAV has caused the yearly epidemics and multiple pandemics over the past century. Thus, it poses a massive threat to public health and results in significant economic losses (Taubenberger and Morens, 2008; Neumann et al., 2009; Taubenberger et al., 2019). Influenza A virus is a single- and negative-stranded RNA virus with eight RNA segments (PB2, PB1, PA, NP, HA, NA, M, and NS). Each viral RNA (vRNA) segment is encapsidated by multiple nucleoprotein (NP) molecules and is also associated with a single, trimeric polymerase complex that comprises polymerase basic protein 1 (PB1), polymerase basic protein 2 (PB2), polymerase acidic protein (PA). Each subunit of vRNA–NP–polymerase is referred to as a viral ribonucleoprotein (vRNP) complex (Eisfeld et al., 2015; Te Velthuis and Fodor, 2016).

The transcription and replication of the IAV genome are performed by the viral RNA-dependent RNA polymerase that is compacted into the vRNP complex. The vRNP complex plays a crucial role in the viral life cycle, viral genome replication, and expression (Noda and Kawaoka, 2010; Resa-Infante et al., 2010; Moeller et al., 2012; Eisfeld et al., 2015). Unlike many other single-stranded RNA viruses, for which the replication cycle is confined to the cytoplasm, influenza viral RNA synthesis takes place in the host cellular nucleus. After viral entry and release of eight vRNPs into the cytoplasm, the vRNPs are transported into the nucleus for the primary transcription and replication that results in the production of both viral messenger RNAs (mRNAs) and complementary RNAs (cRNAs) that serve as templates for vRNAs production. Then the mRNAs are exported into the cytoplasm for protein translation in ribosomes. The newly formed viral proteins, such as PB2, NP monomers, and PA-PB1 heterodimers, traffic back into the nucleus for genome replication and progeny RNPs production. With the assistance of M1 and NEP proteins, progeny vRNPs are exported to cytoplasm and subsequently transported into the cell membrane for the progeny virions assembly and budding. Finally, the released progeny virions infect adjacent cells for a new life cycle (Eisfeld et al., 2015; Peacock et al., 2019).

Due to the limited genome capacity, vRNPs rely on host factors to traffic in and out of the nucleus for efficient viral genome transcription and replication. Following viral entry, internalization, and release of vRNP in the cytoplasm, vRNPs were transported into the nucleus through the nuclear pore complex (NPC) by importin-α:β heterodimer (O'Neill et al., 1995). Importin-α recognizes the nuclear localization sequences (NLSs) on NP and associates with importin-β (Gorlich et al., 1995; Wu et al., 2007; Wu and Pante, 2009). After primary transcription and translation, newly formed polymerase subunits need to form a catalytically active hetero-trimer in the nucleus. It has been shown that newly synthesized PB2 enters into the nucleus by its NLS binding of importin-α isotypes, such as importin-α1 (IMP-α1), importin-α3 (IMP-α3), importin-α5 (IMP-α5), and importin-α7 (IMP-α7) (Resa-Infante et al., 2008), and PA and PB1 translocate to nucleus by the binding of β-importin Ran-binding protein 5 (RanBP5; also known as importin-5 and karyopherin-β3) (Deng et al., 2006). Factors that affect the nuclear import of ribonucleoprotein (RNP) tend to have effects on viral replication, such as Heat Shock Protein 90 (HSP90) (Naito et al., 2007b), Phospholipid scramblase 1 (PLSCR1) (Luo et al., 2018), translation elongation factor 1 delta (eEF1D) (Gao et al., 2020), and LYAR (Yang et al., 2018).

Besides, the transportation of vRNP relies on the host nuclear machineries. NPC is a part of nuclear machinery, and its function coordinates the bidirectional transport of macromolecules between the cytoplasm and nucleus (Terry and Wente, 2007; Lim et al., 2008; Hampoelz et al., 2019; Lin and Hoelz, 2019). It is composed of multiple copies of ~30 different proteins termed nucleoporins (NUPs) to form a basket-like structure (Beck and Hurt, 2017). Nucleoporins have also been reported to play roles in influenza virus replication, such as NUP98, NUP93, NUP214, and NUP153 (Satterly et al., 2007; Muhlbauer et al., 2015; Furusawa et al., 2018; Li et al., 2020; Senbas Akyazi et al., 2020). As a part of the Nup107-160 complex, NUP85 (also known as FROUNT) is required for NPC assembly and maintenance (Harel et al., 2003). It has been found to interact with HIV Tat protein in the cellular nucleus (Brass et al., 2008; Gautier et al., 2009), be involved in inflammation (Terashima et al., 2020), be associated with the chemokine receptor CCR2 and CCR5 to regulate chemokine signaling (Zannettino et al., 2008; Toda et al., 2009, 2014) and tumor progression (Terashima et al., 2020). However, the association of NUP85 with influenza virus infection and its role in influenza virus replication and its mechanisms have not been investigated.

In this study, we found that NUP85 interacted with IAV PB1 and PB2 proteins. Meanwhile, the knockdown of NUP85 delayed the nuclear transportation of vRNP, hindered IAV polymerase activity, and significantly hampered the replication of various subtypes of influenza viruses. Further investigation indicated that NUP85 facilitated the nuclear import of vRNP by assisting the interactions of PB1 with nuclear transporter factor RanBP5, and PB2 with nuclear transporter factor IMP-α1 and IMP-α7. We also found that NUP85 mRNA and protein levels were reduced in A549 cells upon IAV infection. Our results strongly demonstrate that NUP85 is a host factor essential for influenza A virus infection.

## Materials and methods

### Cells and viruses

Human alveolar adenocarcinoma epithelial (A549) cells, human embryonic kidney (HEK 293 T) cells, and Madin–Darby canine kidney (MDCK) cells were used in this study. A549 cells, MDCK cells and HEK 293 T cells were cultured in Ham’s F-12 medium, Dulbecco’s modified Eagle’s medium (DMEM), and minimum essential medium (MEM), respectively. The media were purchased from Thermo Fisher (Waltham, MA, United States), and were supplemented with 10% fetal bovine serum (FBS; ExCell Biology, shanghai, China), 100 U/ml penicillin, and 0.1 mg/ml streptomycin. All cells were cultured at 37°C in a 5% CO_2_ humidified incubator.

The IAVs used in experiments were Influenza A/Puerto Rico/8/1934 (H1N1), A/Zhejiang /163/2020 (H3N2), and A/swine/Jiangsu/C1/2008 (H9N2). All the viruses were propagated in 10-day-old specific-pathogen-free (SPF) chicken embryos, and viral titers were determined by calculating TCID_50_ per milliliter using the Reed–Muench method in MDCK cells.

### Antibodies and reagents

Antibodies used for Western blot, immunoprecipitation, and indirect immunofluorescence were anti-NUP85 rabbit polyclonal antibody (catalogue No. 19370-1-AP, Proteintech, Rosemont, United States), anti-Flag mouse monoclonal antibody (catalogue No. 66008-3-Ig, Proteintech, Rosemont, United States), anti-IAV NS1 rabbit polyclonal antibody (catalogue No. GTX125990, GeneTex, CA, United States); anti-HA rabbit polyclonal antibody (catalogue No. 51064-2-AP, Proteintech, Rosemont, United States), anti-GAPDH mouse monoclonal antibody, goat anti-rabbit HRP and goat anti-mouse HRP (catalogue No. FD0063, FDR007, and FDM007, FD bio, Hangzhou, China), Tritc Conjugated goat mouse polyclonal antibody (catalogue No. HA1017, HuaBio, Hangzhou, China), anti-Histone H3 mouse monoclonal antibody (catalogue No. EM30605, HuaBio, Hangzhou, China) and Alexa Fluor 488 conjugated goat rabbit polyclonal antibody (catalogue No. HA1121, HuaBio, Hangzhou, China), Anti-Flag M2 beads (Sigma, MO, United States) and DAPI (Beyotime, Shanghai, China).

### Plasmids, small interfering RNAs and transfection

The nucleic acid for wild-type NUP85 (Nucleoporin 85, GenBank accession No. NM024844.5), RanBP5 (IPO5 importin 5, GenBank accession No. NM002271.6), and the full-length open reading frames (ORFs) of human importin α1 (GenBank accession No. BC005978.1), importin α3 (GenBank accession No. AK291041), importin α5 (GenBank accession No. CR456743.1), and importin α7 (GenBank accession No. AF060543) were obtained from the A549 cells by RT-PCR and subsequently subcloned into eukaryotic expression vector pCMV-HA at EcoRI and KpnI sites, respectively. The PA (GenBank accession No. CY147539), PB1 (GenBank accession No. CY147540), PB2 (GenBank accession No. CY147541), and NP (GenBank accession No. CY147537) of the PR8 H1N1 virus were amplified from viral supernatant and cloned into the vector pCMV-Flag at HindIII and KpnI sites, respectively. All plasmid constructs were confirmed by sequencing.

Small interfering RNAs targeting NUP85 (siRNA1 and siRNA2) and a validated negative control siRNA (NC siRNA) were purchased from GenePharma (Shanghai, China). The knockdown efficiency was examined by qRT-PCR. The primer sequences of PCR for plasmids construction are listed in Table 1, and the sequences for siRNAs are listed in Table 2.

**Table 1:** List of primers used for PCR in this study.

Target gene	Sequence(5′ to 3′)
HA-NUP85	F-ATGGAGGAGCTCGATGGCGA
	R-TCAGGAACCTTCCAGTGAGCC
Importin α1	F-ATGTCCACCAACGAGAATGCTAATACACCAGCTGCC
	R-GGTACCTAAAAGTTAAAGGTCCCAGGAGCCCCATCC
Importin α3	F-ATGGCGGACAACGAGAAACTGGACAACCAACG
	R-CGGTACCTAAAACTGGAACCCTTCTGTTGGTACATTGGC
Importin α5	F-ATGACCACCCCAGGAAAAGAGAACTTTCGCCT
	R-CTTAAAGCTGGAAACCTTCCATAGGAGCCTCACA
Importin α7	F-ATGGAGACCATGGCGAGCCCAGGGAAAGACAATTA
	R-TTATAGCTGGAAGCCCTCCATGGGGGCCTCA
RanBP5	F-ATGGCGGCGGCCGCGGCGGAGCAGCAACAGTTC
	R-TCACGCAGAGTTCAGGAGCTCCTGAATGGCGGCCTGC

**Table 2:** List of sequences for siRNAs used in this study.

siRNAs	Sequence(5′ to 3′)
siRNA1	F-GGGUCGAUUACUUUGAUUATT
	R-UAAUCAAAGUAAUCGACCCTT
siRNA2	F-GAGCAUGUAUGGAGGAAAUTT
	R-GGAGGAGTGGGTGTCGCTG

Transfection of Plasmids and siRNAs to HEK293 cells was performed using GeneTwin (Biomed, Beijing, China), DMEM medium without serum and antibiotics. Briefly, plasmids, siRNAs, and GeneTwin were diluted to equal volumes with DMEM and incubated for 5 min at room temperature. The diluted GeneTwin and the diluted DNA (or RNA) were mixed and incubated for 10 min at room temperature, and then the mixture was added to the cells. Transfection of siRNA in A549 cells was mediated by jetPRIME (Polyplus, California, United States) according to the manufacture’s instruction. Four hours post-transfection, transfection reagents were replaced with medium containing 10% FBS.

### Virus infection and titration

When the A549 cells in 12-well plates grew to ~95% confluent monolayer, the cell culture medium was withdrawn, and the cells were washed twice with phosphate-buffered saline (PBS). Then viruses in the viral growth medium, which is the medium supplemented with 2% BSA (Sigma, MO, United States) and 2 μg/ml of tosylsulfonyl phenylalanyl chloromethyl ketone (TPCK)-trypsin (Sigma, MO, United States), were inoculated in the cells at indicated MOIs. After adsorption at 37°C for 1 h, the inoculum was removed and replaced with a viral growth medium. And viral supernatants were harvested at the indicated time points post-infection.

To measure the viral titers in the supernatant, we plated MDCK cells in 96-well plates and let them grow to 90%–95% confluence overnight. The supernatant was diluted serially in MEM supplemented with 2% BSA and 2 μg/ml of TPCK-trypsin and inoculated into the cells with PBS washed twice. The IAV-induced cytopathic effect (CPE) was monitored for 24–96 h. TCID_50_ was then calculated by the Reed-Muench formula.

### RNA isolation and qRT-PCR

A two-step real-time quantitative RT-PCR was used to examine specific mRNA levels. Cells were lysed for quantitative reverse transcription-PCR (qRT-PCR), and total RNA was extracted according to the manufacturer’s instructions (Easy-do Bio, Zhejiang, China). Reverse transcription was carried out with HiScript Q Select RT SuperMix for qPCR (+gDNA wiper; Vazyme, Nanjing, China). To detect the viral NP mRNA, we used oligo(dT) primer for the reverse transcription. And to detect the viral NP cRNA and vRNA, we used primer specifically targeting IAV H9N2 JSC1 cRNA and vRNA for reverse transcription. Quantitative PCR was performed using ChamQ Universal SYBR qPCR Master Mix (Vazyme, Nanjing, China), and run on an Mx3005P quantitative PCR system(Agilent, California, US). The primers of RT-PCR were designed using PrimerQuest Tool. The sequences of primers for qRT-PCR are listed in Table 3. Each gene was amplified in triplicate, and the mean threshold (Ct) values were calculated. GAPDH was used for normalization in gene expression analysis. Relative fold changes in gene expression among groups were determined using the 2^−ΔΔCt^ method.

**Table 3:** List of primers for qRT-PCR assay in this study.

Target genes	Sequence(5′ to 3′)
GAPDH	F-GCTAAGGCTGTGGGCAAGG
	R-GGAGGAGTGGGTGTCGCTG
NUP85	F-GCCAACAGTCACTTTGATTCC
	R-CACATACCAGCATCTCCCCTG
NS1	F-TCGAAACAGCTACTCGTGCG
	R-ACTGTGAAGCAGGCACAGAA
18 s RNA	F-AGTTGGTGGAGCGATTTGT
	R-TGAGCCAGTCAGTGTAGCG
NP	F-CAAAGAGGAGATCAGGAGGA
	R-TTCCAGTACGCACGAGAGCT

### Western blot assay

Cells were collected and washed with PBS and then lysed with RIPA lysis buffer (Beyotime, Shanghai, China) containing Phenylmethanesulfonylfluoride (PMSF, Beyotime, Shanghai, China). The cell lysates were centrifuged at 12,000 g for 10 min at 4°C, and the supernatant was collected, then added with SDS-PAGE sample loading buffer (Beyotime, Shanghai, China) and boiled for 10 min. Equal amounts of protein samples were subjected to SDS-polyacrylamide gel (FD Bio, Hangzhou, China) electrophoresis and transferred to PVDF membrane (Millipore, Darmstadt, Germany). The membrane was probed with various primary antibodies as indicated and detected using the ECL system (Beyotime, Shanghai, China and Thermo Fisher, Massachusetts, United States) with alkaline phosphatase-conjugated secondary antibodies according to the manufacturer’s protocol.

### Cell viability assay

To detect the effect of NUP85 silencing on cell proliferation, we measured the cell viability of A549 cells transfected with NUP85 siRNA1 or NC siRNA by Cell Counting Kit-8 (CCK-8) activity according to the manufacturer’s instructions (APE Bio, Texas, United States). In brief, different amounts of cells in 96-well plates were transfected with NUP85 siRNA1 or NC siRNA, and cell viability was measured at 36 h post-transfection. The CCK-8 reagent was added to each well of the 96-well plates, and cells were incubated at 37°C for 2 h; the absorbance at 450 nm was measured by a microplate reader (Bio-Tek, Vermont, United States).

### Minigenome assay

The minigenome assay was performed as described previously (Zhou et al., 2011). We firstly knocked down NUP85 expression by small interfering RNAs treatment for 24 h, and then we transfected HEK293T cells with 0.2 ug of pPoll-NS-luc plasmid that contains the firefly luciferase gene flanked by the non-coding regions of influenza nonstructural (NS) gene segment and 0.5 ug of each of the reverse genetic plasmids encoding PR8 PB2, PB1, PA, and NP by using 30 ul transfection reagent (GeneTwin). Simultaneously, 50 ng of the Renilla luciferase plasmid pRL-TK (Promega) was co-transfected as a transfection efficiency control. At 24 and 48 h post-transfection, the relative polymerase activity (firefly normalized to Renilla) was measured using the dual-luciferase assay kit (Vazyme, Nanjing, China) according to the manufacturer’s instructions. The polymerase activity in the cells co-transfected with NC siRNA was set as 100%, and the activity in the cells co-transfected with NUP85 siRNA was determined relative to that in cells with NC siRNA. The results shown are from three independent experiments performed in triplicate.

### Indirect immunofluorescence assay and confocal microscopy

A549 cells were grown on coverslips to 50% confluence and were transfected with 2 μg of small interfering RNAs. After 24 h, cells were infected with an indicated influenza A virus for 3, 6, and 9 h. Then, cells were fixed with 4% paraformaldehyde (PFA) at 4°C overnight. The next day, cells were washed with PBS three times and permeabilized using 0.2% (vol/vol) Triton X-100 for 15 min, then blocked with 2% (wt/vol) bovine serum albumin (BSA) in PBS for 2 h. Coverslips were incubated with specific antibodies for NUP85 and NP at 4°C for 2 h. After washing with PBS, cells were incubated with indicated Alexa Fluor conjugated secondary antibodies for 2 h at room temperature. After washing the secondary antibodies, nuclei were stained with DAPI for 15 min at room temperature. These samples were observed by confocal microscope (IX81-FV1000; Nikon, Tokyo, Japan) after washing with PBS three times.

### Immunoprecipitation and RNase treatment

HEK293T cells were transfected with the 4 μg of small interfering RNAs for 24 h, and then the cells were transfected with 4 μg plasmids encoding the RNP subunits using GeneTwin (Biomed, Beijing, China) according to the manufacturer’s instructions. After 24 h transfection, the cells were lysed with RIPA lysis buffer (Beyotime, Shanghai, China) with Phenylmethanesulfonylfluoride (PMSF, Beyotime, Shanghai, China) supplement. Then the supernatant was collected and used to set up immunoprecipitation assay. For RNase treatment, 100 μg/ml RNase was added to the cell lysate, and the mix was incubated at 37°C for 45 min. Cell extracts were mixed with anti-Flag M2 Magnetic Beads (Sigma, MO, United States) and incubated at 4°C for overnight. The next day, the beads were washed thrice with PBS, and protein-antibody complexes were eluted in buffer (20 mM Tris; 150 mM NaCl; 0.15% HCl; 0.05% Tween). The immunoprecipitated proteins and remaining cell lysates were separated on SDS-PAGE followed by transferring to nitrocellulose for Western blot.

### Nuclear and cytoplasmic protein fractionation

HEK293T cells treated with small interfering RNAs for 24 h were transfected with Flag-PB2 or co-transfected with Flag-PB1 and PA plasmids using GeneTwin (Biomed, Beijing, China) according to the manufacturer’s instructions. After 24 h post-transfection, cells were harvested with PBS and collected by centrifugation at 1,000 rpm for 5 min. Nuclear and Cytoplasmic Protein Extraction Kit (Beyotime, Shanghai, China) was used to prepare nuclear and cytoplasmic lysates. For protein isolation, the whole lysates and the cytoplasmic and nuclear fractions were added with SDS-PAGE sample loading buffer (Beyotime, Shanghai, China) and boiled for 10 min, followed by Western blot analysis.

### Cytoplasmic and nuclear RNA fractionation

HEK293T cells treated with small interfering RNAs for 24 h were infected with influenza A viruses. At 6 and 12 h post-infection, cells were collected and washed with PBS three times and then lysed with 0.5% Nonidet P-40 (Sangon Biotech, Shanghai, China) containing RNase Inhibitor and DL-Dithiothreitol (Beyotime, Shanghai, China). The cell lysates were centrifuged at 12,000 g for 5 min at 4°C. The supernatant was collected for cytoplasmic RNA extraction and the precipitate was collected for nuclear RNA extraction.

### Statistical analysis

All data were expressed as mean ± standard deviation (SD) from at least three independent experiments. The statistical analyses were performed with a two-tailed Student’s *t*-test and a two-way ANOVA test. Differences between groups were considered significant if *p* < 0.05 (indicated with *), highly significant if *p* < 0.01(indicated with **) and extremely significant if *p* < 0.001 (indicated with ***).

## Results

### Knockdown of NUP85 suppresses IAV replication

**Figure 1:**
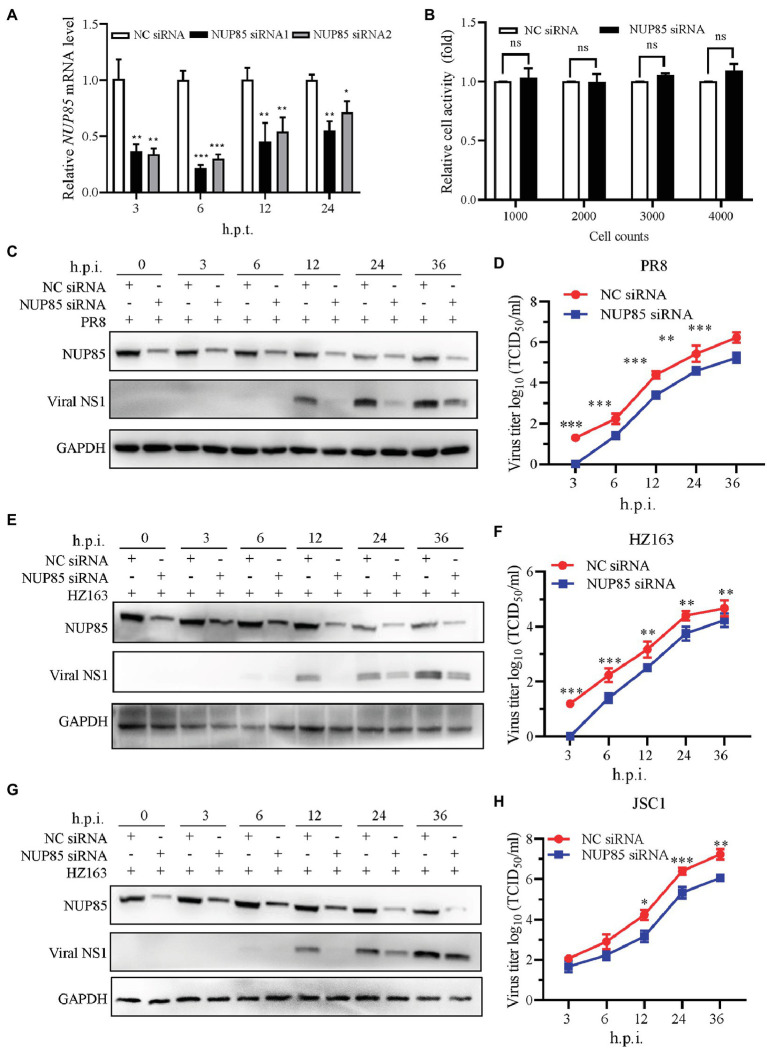
NUP85 knockdown suppresses IAV replication. **(A)** The silencing efficiency of *NUP85*-specific siRNAs. A549 cells were transfected with two individual siRNAs targeted to *NUP85* (NUP85 siRNA1 and NUP85 siRNA2) or non-target siRNA as negative control (NC siRNA), and the cells were harvested for RNA isolation at 3, 6, 12, and 24 h.p.t., followed by qRT-PCR to detect the relative expression level of *NUP85*. **(B)** The effect of NUP85 siRNA1 on A549 cell viability. Indicated numbers of A549 cells were transfected with NUP85 siRNA1 and NC siRNA for 36 h.p.i. Cell viability was measured by Cell Counting Kit-8 (CCK-8) assay. **(C,E,G)** The effect of siRNA on the expression of IAV NS1 protein. A549 cells were transfected with NUP85 siRNA and NC siRNA and then infected with H1N1 PR8, H3N2 HZ163, and H9N2 JSC1 (MOI = 0.1). Whole cell lysates were collected at the indicated time points and subjected to Western blot to detect the NUP85 and viral NS1 protein levels. GAPDH was used as an internal control. Images are from one of three independent experiments. **(D,F,H)** A549 cells were transfected with NUP85 siRNA and NC siRNA and then infected with influenza A virus JSC1, PR8 and HZ163 (MOI = 0.01). Cell supernatants were harvested at 3, 6, 12, 24, and 36 h.p.i. Virus titers were determined by TCID_50_ assay on MDCK cells. Data are mean ± SD of three independent experiments. Significance is calculated by unpaired *T*-test; *indicates *p* < 0.05; **indicates *p* < 0.01; *** indicates *p* < 0.001.

Previous analysis has shown that NUP85 potentially plays a role in influenza virus replication (Li et al., 2020). To determine the role of NUP85 during IAV infection, we applied small interfering RNAs (siRNA) to knockdown NUP85 in A549 cells. Firstly, we confirmed the knockdown efficiency by measuring the NUP85 mRNA level by qRT-PCR. As shown in Figure 1A, expression of NUP85 in the A549 cells that were transfected with two different siRNAs targeted to NUP85 (NUP85 siRNA1 and NUP85 siRNA2) was reduced by 70% ~ 80% as compared to the cells that were transfected with negative control RNAi (NC siRNA) at 6 h post-transfection (h.p.t.). The silencing efficiency went down over time as the degradation of siRNA happened in the cells, but still, the NUP85 expression level was reduced by 40 ~ 70% of control cells at 12 and 24 h.p.t. (Figure 1A). We selected the NUP85 siRNA1, whose silencing efficiency was a little higher than NUP85 siRNA2 for the following experiments. Also, the cell viability was measured by Cell Counting Kit-8 (CCK-8) assay, and the results showed that knockdown of NUP85 by NUP85 siRNA transfection does not have a cytotoxic effect on A549 cells (Figure 1B).

To examine the function of NUP85 during virus replication, we infected A549 cells with influenza virus A/Puerto Rico/8/1934 (H1N1) (PR8), A/swine/Jiangsu/C1/2008 (H9N2) (JSC1), A/Zhejiang/163/2020 (H3N2) (HZ163) after NUP85 siRNA or NC siRNA transfection. Samples were collected at 0, 3, 6, 12, 24, and 36 h post-infection (h.p.i.), and Western blot was performed to detect viral NS1 protein in the infected cells. The result showed much less viral NS1 protein was detected in the cells with NUP85 siRNA treatment compared with NC siRNA treatment at different time points post-infection by three different strains of viruses (Figures 1C,E,G). To further confirm the impact of NUP85 knockdown on IAV replication, we determined the growth kinetics of H1N1 PR8, H3N2 HZ163, and H9N2 JSC1 in NUP85 siRNA- or NC siRNA-treated A549 cells. Supernatant samples were collected at 3, 6, 12, 24, and 36 h.p.i., and the viral titers were examined by 50% tissue culture infective dose (TCID_50_) assay in MDCK cells. The results showed that the viral loads of H1N1 PR8, H3N2 HZ163, and H9N2 JSC1 were significantly decreased in NUP85 siRNA-treated cells compared to NC siRNA-treated cells (Figures 1D,F,H). Taken together, these results showed that the knockdown of NUP85 suppressed IAV replication.

### Knockdown of NUP85 inhibits polymerase activity and suppresses RNA synthesis during IAV infection

**Figure 2:**
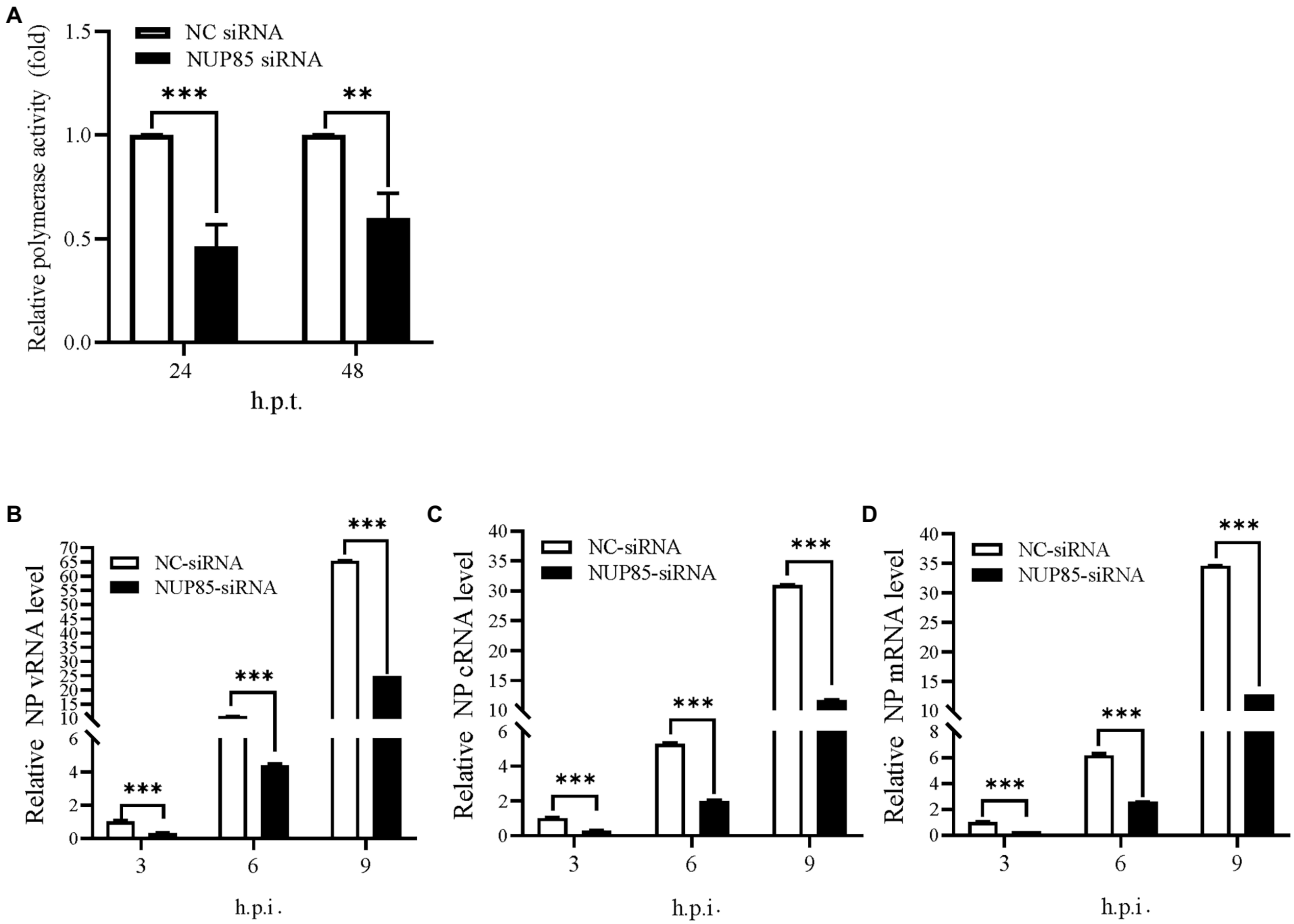
Knockdown of NUP85 inhibits polymerase activity and suppresses RNA synthesis during IAV infection. **(A)** HEK293T cells treated with NUP85 siRNA or NC siRNA were transfected with vRNP reconstitution plasmids together with Renilla plasmids. Luciferase activities were measured at 24 and 48 h.p.t., and Renilla luciferase was used as an internal control. **(B–D)** A549 cells were transfected with NUP85 siRNA and NC siRNA for 24 h and then infected with influenza A virus JSC1 (MOI = 2). Cells were harvested for RNA isolation at 3, 6, and 9 h.p.t., followed by qRT-PCR to detect vRNA, cRNA and mRNA relative expression level of NP. The viral RNA levels were normalized to the 18S rRNA level. Data are mean ± SD of three independent experiments. Significance is measured by unpaired *T*-test; ** indicates *p* < 0.01. *** indicates *p* < 0.001.

IAV uses an RNA-dependent RNA polymerase (RdRp) compacted into RNP to transcribe and replicate its RNA genome. To investigate whether deduction of viral replication in NUP85 knockdown cells would result from the decreased polymerase activity, we performed a well-established mini-replicon assay to examine the effect of NUP85 on the polymerase activity of the H1N1 PR8 virus (Lutz et al., 2005). In the assay, HEK293T cells treated with NUP85 siRNA or NC siRNA were transfected with plasmids encoding RNP subunits PB1, PB2, PA, and NP, as well as a Poll-driven RNA expression plasmid encoding the firefly luciferase gene which was franked by viral NS non-coding sequences. The experiment showed that knockdown of NUP85 resulted in an approximately 55% reduction in the polymerase activity at 24 h.p.t. and ~40% reduction in the polymerase activity at 48 h.p.t. compared with control. The lowered deduction of polymerase activity at 48 h.p.t. may result from the decreased silencing efficiency along with the degradation of the interfering RNA (Figure 2A). The result indicates that NUP85 silencing reduces viral polymerase activity, thereby inhibiting IAV replication.

To further determine whether NUP85 knockdown affected viral genome replication, A549 cells were transfected with the NC siRNA and NUP85 siRNA, followed by infection of H9N2 JSC1 at an MOI of 2. Cells were collected, and viral RNAs were examined at 3, 6, and 9 h post-infection. The levels of viral RNAs (vRNA, cRNA, and mRNA) were considerably lower in the NUP85 siRNA-treated cells than those in NC siRNA-treated cells (Figures 2B–D). The results indicated that NUP85 is essential in viral RNA synthesis.

### Knockdown of NUP85 hinders the vRNP import into nucleus

**Figure 3:**
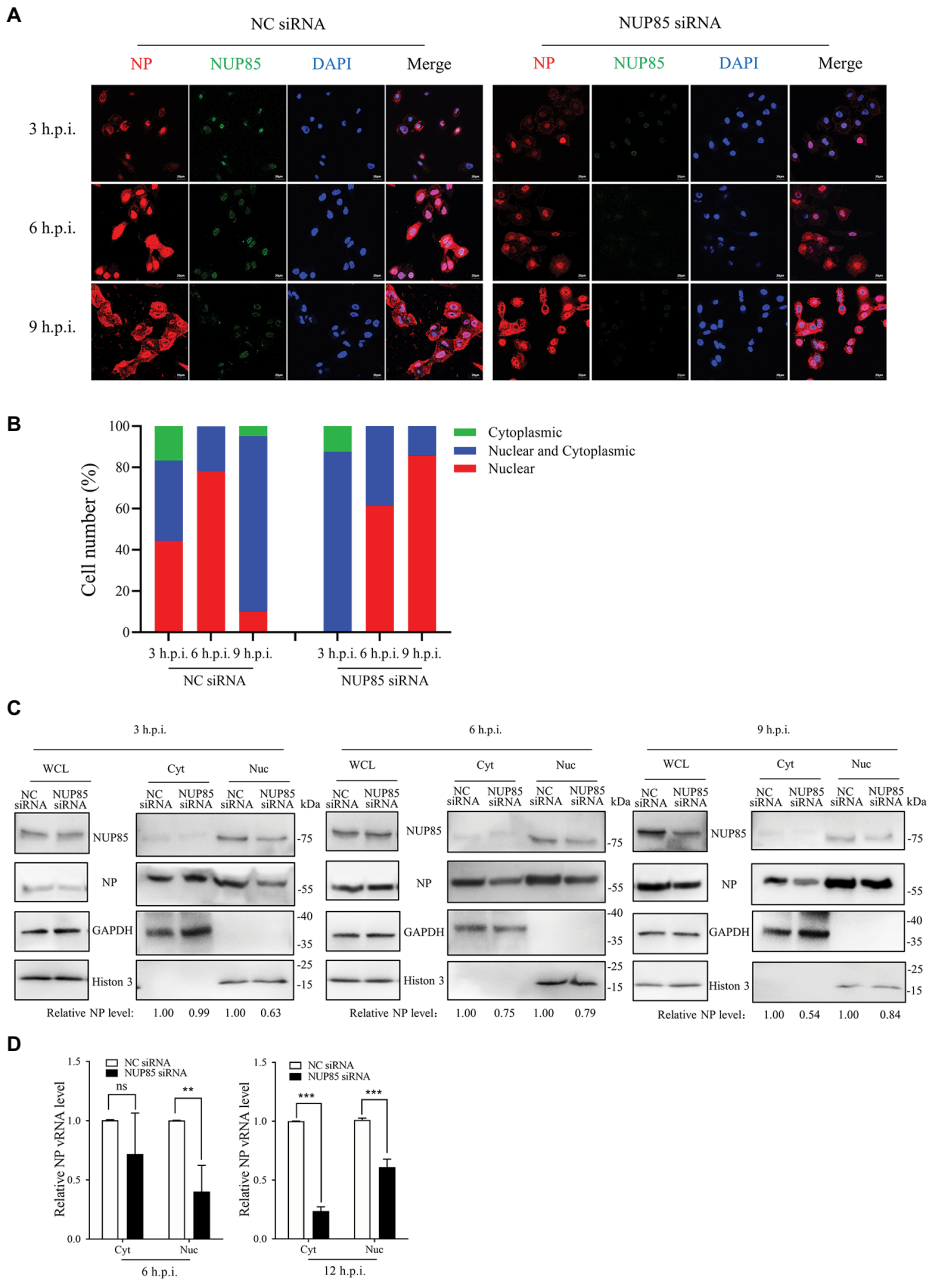
Knockdown of NUP85 inhibits the nuclear import of vRNP. **(A)** Confocal microscopy analysis of the nucleocytoplasmic distribution of vRNP in virus-infected NUP85 knockdown cells. A549 cells were treated with NUP85 siRNA or NC siRNA and then were infected with the H9N2 JSC1 virus (MOI = 3). At 3, 6, and 9 h post-infection, cells were fixed, permeabilized, and stained with mouse anti-NP (red), rabbit anti-NUP85 (green) and DAPI (blue). The scale bar represents 20 μm. Images are representative of three independent experiments. **(B)** Quantitative analysis of vRNP localization in the infected cells was performed by calculating cell numbers with related fluorescence. At least 100 cells in each group were scored. **(C)** Western blot analysis of the distribution of NP in the cytoplasmic and nuclear fractions in virus-infected NC siRNA and NUP85 siRNA-treated cells. A549 cells were treated with NUP85 siRNA or NC siRNA and then were infected with the H9N2 JSC1 virus (MOI = 3). Cells were harvested at 3, 6, and 9 h.p.i. Then, the nucleocytoplasmic distribution of NP was examined by Western blot and grayscale analysis. The relative protein level of NP in nucleus is indicated with its gray intensity divided by that of Histone 3, and the relative protein level of NP in cytoplasmic is indicated with its gray intensity divided by GAPDH. Then they are normalized by that of control group, the results of which are shown at the bottom panel. Images are representative of three independent experiments. **(D)** qRT-PCR analysis of the distribution of viral vRNA in the cytoplasmic and nuclear fractions in virus-infected NC siRNA and NUP85 siRNA-treated cells. A549 cells were treated with NUP85 siRNA or NC siRNA and then were infected with the H9N2 JSC1 virus (MOI = 3). Cells were harvested and the cytoplasmic and nuclear parts were fractionated at 6, and 12 h.p.i., followed by qRT-PCR to detect the relative level of NP vRNA. Data are mean ± SD of three independent experiments. Significance is measured by unpaired *T*-test; ns, no significance. ** indicates *p* < 0.01. *** indicates *p* < 0.001.

Transcription and replication of influenza A viral gene happen in the nucleus, so the nuclear import of vRNP is pivotal to the transcription and replication of influenza A viral gene. We hypothesized that NUP85 knockdown might affect the nuclear import of vRNPs in A549 cells. Viral NP is a major component of the vRNP complex, and it mediates the nuclear import of the vRNP complex *via* its nuclear localization signals (NLSs) (Naito et al., 2007a; Kawaguchi et al., 2011; Luo et al., 2018). We examined the cellular distribution of vRNP by imaging viral NP in A549 cells. A549 cells treated with NUP85 siRNA or NC siRNA were infected with H9N2 JSC1, and then viral NP and nucleus were stained to display the vRNP subcellular localization by immunofluorescence microscopy. In NC siRNA-treated cells, NP had clearly accumulated exclusively in the nucleus of approximately 45% of cells, and it accumulated both in the nucleus and cytoplasm of roughly 40% of cells at 3 h.p.i., and then it was predominantly located in the nucleus (81% of the cells) at 6 h.p.i. (Figures 3A,B). While none of the cells treated with NUP85 siRNA had NP exclusively localized in the nucleus at 3 h.p.i., and most NP was still distributed in the cytoplasm at the time point. At 6 h.p.i., the percentage of exclusive nuclear localization of viral NP in the NUP85 knockdown cells decreased over 20% compared with control cell (Figures 3A,B). After the primary transcription of the viral genes, the newly synthesized RNP subunit mRNA was exported from the nucleus to the cytoplasm for protein translation to form more RNPs (Matsuoka et al., 2013). At a later stage (9 h.p.i.), NP started to re-localization into the cytoplasm in NC siRNA-treated cells, but it still predominantly localized in the nuclear in the NUP85 siRNA-treated cells. These results suggest that knockdown of NUP85 hinders the nuclear import of vRNP, which leads to decreased polymerase activity and reduced viral replication.

To further confirm the defective nuclear import of vRNP when NUP85 was knocked down, we fractionated the cytoplasm and nucleus of infected cells and examined vRNP distribution indicated by NP expression at 3, 6, and 9 h post-infection by Western blot. As shown in Figure 3C, the nuclear distribution of vRNP decreased by 37% at 3 h.p.i. in the NUP85-knockdown cells compared with the control cells. The number changed to 21% as more vRNPs entered into the nucleus at 6 h.p.i., but the total amount of vRNP in the nuclear and cytoplasm is less in the NUP85-knockdown cells than in the control cells. At a later stage (9 h.p.i.), there is only one-third of vRNPs distributed in the nucleus in control cells, and there are still nearly a half vRNP in the nucleus in the NUP85 knockdown cells. The results are consistent with what we have seen in the immunofluorescent microscopy assay (Figures 3A,B). Furthermore, we examined vRNA levels in the cytoplasmic and nuclear fractions at 6 and 12 h post-infection by qRT-PCR. The NP vRNA in the nucleus in the NUP85 knockdown cells was only around a half when compared with the control cell at 6 and 12 h post-infection. The NP vRNA in the cytoplasm in the NUP85 knockdown cells was only 23% compared to that in the control cell at 6 h post-infection (Figure 3D). And the total vRNA in the nucleus and cytoplasm decreased by 45% in the NUP85 knockdown cells compared to that in the control cells at 6 h post-infection, and it decreased by 60% in the NUP85 knockdown cells at 12 h post-infection (Figure 3D). The results indicated that the knockdown of NUP85 impaired nuclear import of vRNP and further resulted in the decreased polymerase activity and reduced viral replication.

### NUP85 binds to IAV RNP subunits PB1 and PB2

**Figure 4:**
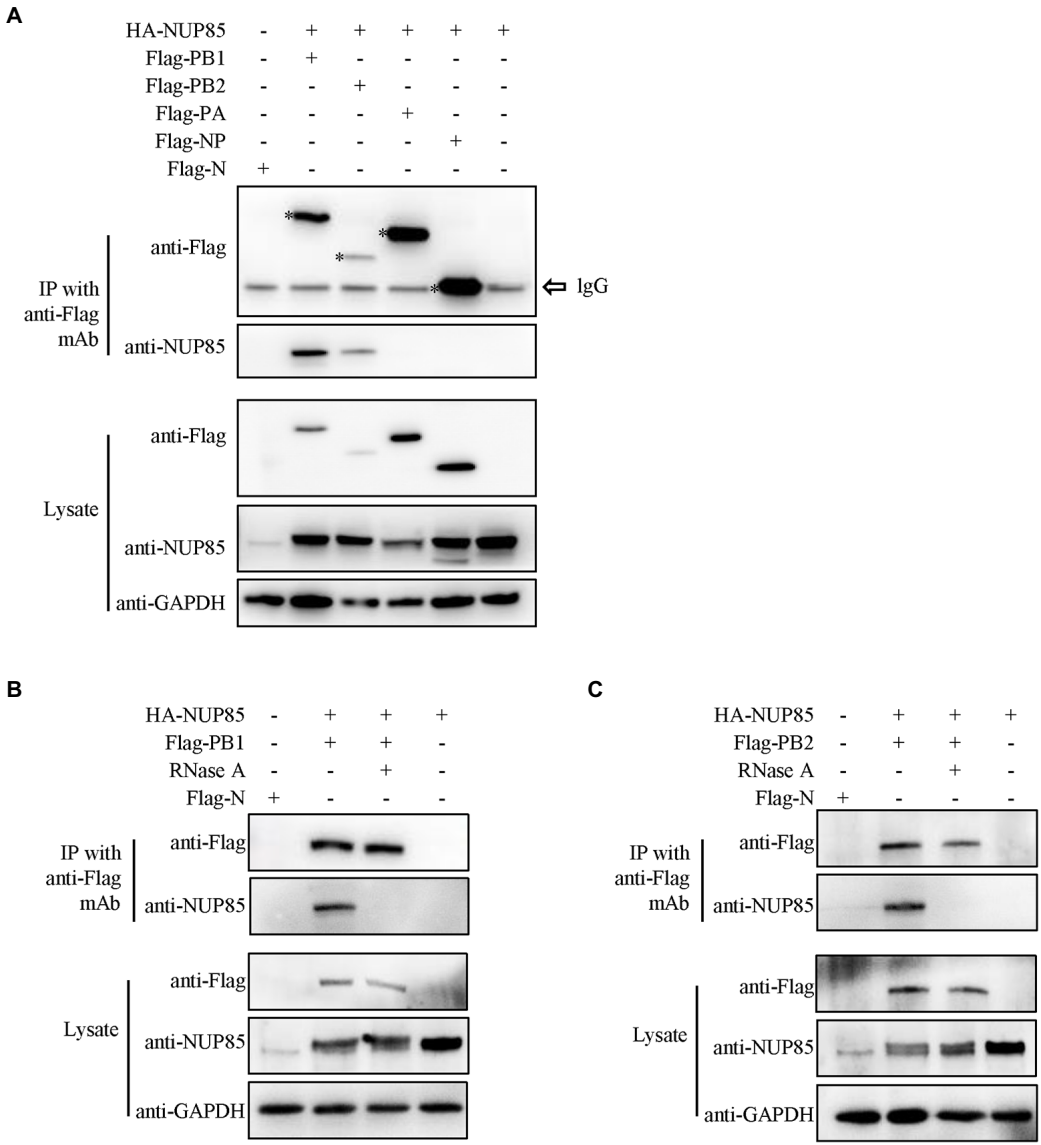
Interactions between NUP85 and RNP subunits. **(A)** HEK293T cells were co-transfected with HA-NUP85 and Flag-PA, Flag-PB1, Flag-PB2, Flag-NP, or Flag-N, respectively. Cells were then lysed at 24 h post-transfection, and immunoprecipitation was performed using anti-Flag antibodies followed by Western blot. **(B)** HEK293T cells were co-transfected with HA-NUP85 with Flag-PB1 and Flag-N or HA-NUP85, respectively. Cells were lysed and treated the RNase at 24 h post-transfection, and immunoprecipitation was performed using anti-Flag antibodies followed by Western blot. **(C)** HEK293T cells were co-transfected with HA-NUP85 with Flag-PB2 and Flag-N or HA-NUP85, respectively. Cells were lysed and treated the RNase at 24 h post-transfection, and immunoprecipitation was performed using anti-Flag antibodies followed by Western blot.

NUP85 is a component of the Nup107-160 subunit of the nuclear pore complex, which is embedded in the nuclear envelope and mediates bidirectional transport of macromolecules between the cytoplasm and nucleus (Beck and Hurt, 2017). IAV vRNP transports into the nucleus by interacting with host factors. To determine the associations between NUP85 and each RNP subunit, Flag-PA, Flag-PB1, Flag-PB2, or Flag-NP and hemagglutinin (HA)-NUP85 were co-transfected into HEK293T cells, respectively. Then, co-immunoprecipitation (co-IP) was performed at 24 h post-transfection. Results showed that NUP85 could co-precipitate with Flag-PB1 and Flag-PB2, but not with Flag-NP and Flag-PA (Figure 4A). Since IAV RNP subunits were the RNA-binding proteins, we also performed the same co-IP assays under RNase A treatment. Results showed that NUP85 was not co-precipitated with Flag-PB1 and Flag-PB2 under the RNase A treatment (Figures 4B,C), suggesting that NUP85 binds to PB1 and PB2 in an RNA-dependent manner.

### Knockdown of NUP85 impedes nuclear import of IAV RNP subunits PB1 and PB2

**Figure 5:**
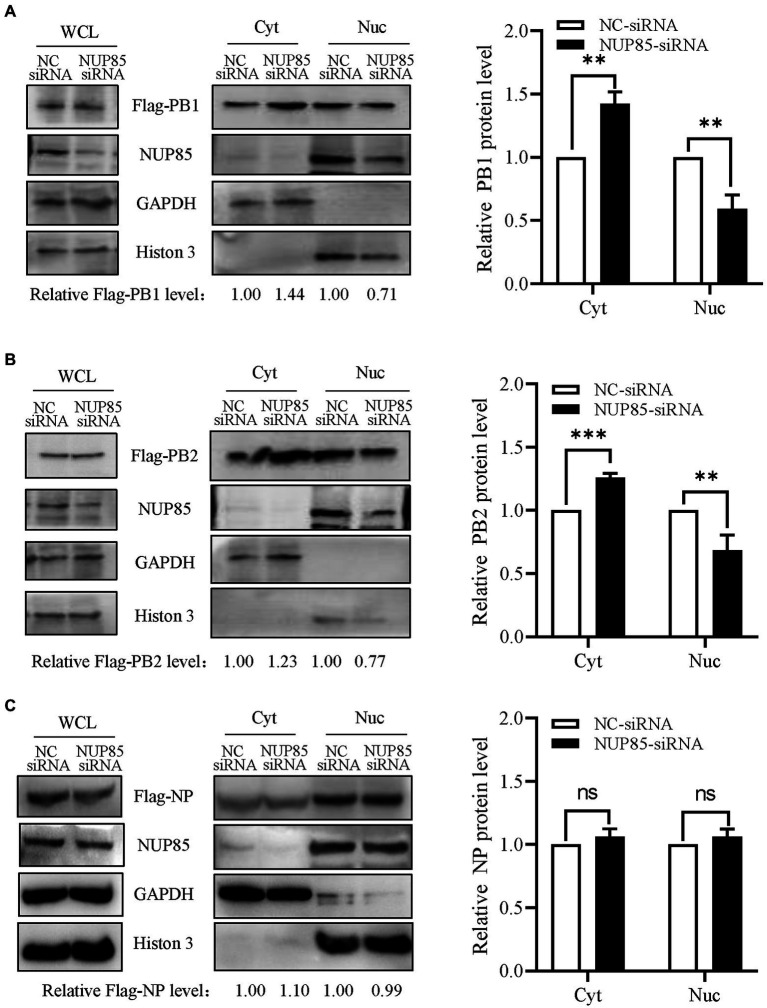
Knockdown of NUP85 affects the nucleocytoplasmic distribution of viral PB1 and PB2, but not NP. **(A)** Plasmids expressing Flag-PB1 and PA were transfected into HEK293T cells treated with NUP85 siRNA or NC siRNA, and the nucleocytoplasmic distribution of Flag-PB1 was examined by Western blot and densitometry analysis. Statistical analysis of PB1 distribution in the NC siRNA-treated or NUP85 siRNA-treated cells was shown on the right. **(B,C)** Plasmids expressing Flag-PB2 **(B)** or Flag-NP **(C)** were transfected into HEK293T cells treated with NUP85 siRNA or NC siRNA, and the nucleocytoplasmic distribution of Flag-PB2 was examined by Western blot and grayscale analysis. The statistical analysis of relative band intensity of PB2 **(B)** or NP **(C)** was shown on the right. Western blot densitometry analysis was performed using software ImageJ. Histone 3 and GAPDH were used as loading controls for nuclear and cytoplasmic fractions, respectively. The relative protein level of PB1, PB2, and NP in nucleus is indicated with its gray intensity divided by that of Histone 3, and the relative protein level of PB1, PB2, and NP in cytoplasmic is indicated with its gray intensity divided by GAPDH. Then they are normalized by that of control group, the results of which are shown at the bottom panels **(A–C)**. Data are mean ± SD of three independent experiments. Significance is measured by unpaired *T*-test; ** indicates *p* < 0.01. *** indicates *p* < 0.001. ns, no significance.

Since NUP85 knockdown hindered the nuclear import of vRNP and the interaction of NUP85 with RNP subunits PB1 and PB2, we further examined the effect of NUP85 on the nuclear import of PB1 and PB2. Since PB1 enters the nucleus with PA as a dimer, A549 cells were treated with NUP85 siRNA or NC siRNA, then transfected with plasmids expressing Flag-PB1 and Flag-PA for 24 h. The results of nuclear and cytoplasmic fractionation showed that the relative protein level of Flag-PB1 in the cytoplasmic was raised by about 44%, and that in the nucleus was decreased by about 29% in the NUP85 siRNA-treated cells compared with the NC siRNA-treated cells (Figure 5A). To examine the nucleocytoplasmic distribution of viral PB2, we transfected Flag-PB2 into the NUP85 siRNA- or NC siRNA-treated cells and determined the PB2 protein levels in cellular nuclear and cytoplasmic parts. Similar to PB1, the relative Flag-PB2 protein level in the cytoplasmic was raised about 23%, and that in the nucleus was decreased by about 23% in the NUP85 siRNA-treated cells compared with the NC siRNA-treated cells (Figure 5B). To further confirm the effect of NUP85 on the nuclear import of IAV RNP subunits PB1 and PB2 is specific, we also examined the nuclear import of IAV RNP subunit PA. PA contains NLS and does not bind with NUP85. As shown in Figure 5C, the knockdown of NUP85 did affect the nuclear import of NP. These results indicate that NUP85 affects the nuclear import of RNP subunits PB1 and PB2, and the process relies on the interaction of NUP85 with PB1 and PB2.

### NUP85 assists the binding of PB1 to RanBP5 and the binding of PB2 to importin α1 and importin α7

**Figure 6:**
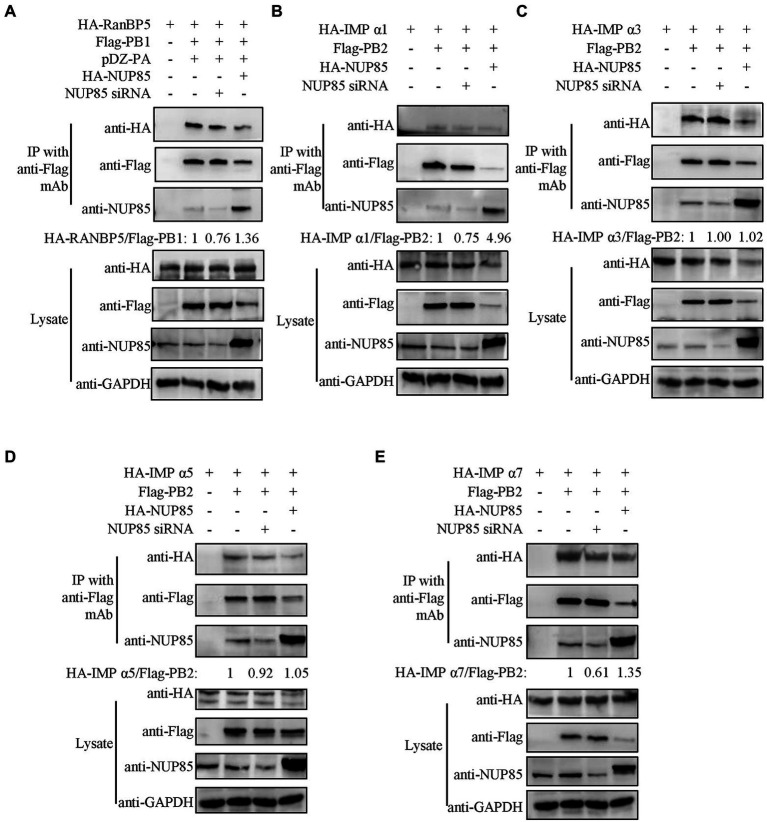
NUP85 facilitates IAV PB1-RanBP5, PB2-importin α1, and PB2-importin α7 interactions. **(A)** Effect of NUP85 on the interaction between RanBP5 in PB1. HEK293T cells were treated with NUP85 siRNA or NUP85 expressing plasmids and then transfected with the indicated plasmids for 24 h. Cell lysates were immunoprecipitated with an anti-Flag antibody. RanBP5 and Flag-PB1 levels were detected *via* Western blot. The band intensities were quantified by ImageJ, and relative precipitated HA-RanBP5 /Flag-PB1 ratios are shown at the bottom. **(B–E)** Interaction of PB2 with importin α family members, importin α1 **(B)**, importin α3 **(C)**, importin α5 **(D)**, and importin α7 **(E)** in the NUP85 siRNA or NUP85 expressing plasmid treated cells. Cell lysates were immunoprecipitated with a mouse anti-Flag antibody. The bound proteins were detected by Western blot with a rabbit anti-HA antibody, a mouse anti-Flag antibody, or a rabbit anti-NUP85 antibody to detect importin α family members, NP, NUP85, and GAPDH, respectively. Images are representative of three independent experiments. The band intensities were quantified by ImageJ. The binding ratio of importin and PB1 or PB2 was calculated with the gray value of the importin divided by that of Flag, and then normalized by the that of control group, the results of which are present at the bottom of the panels. Images are representative of three independent experiments.

Previous studies have already reported that IAV PB1 enters the nucleus *via* a non-classical transport pathway mediated by RanBP5 (Deng et al., 2006; Huet et al., 2010; Hutchinson et al., 2011), and viral PB2 enters the nucleus by binding multiple isoforms of importin α in the classic importin α - importinβ1 pathway (Resa-Infante et al., 2008; Gabriel et al., 2011; Beck and Hurt, 2017). However, unlike PB2, which can accumulate efficiently in the nucleus in the absence of other viral proteins, PB1 and PA can be efficiently imported into the nucleus only after they form a dimer in the cytoplasm (Jones et al., 1986; Smith et al., 1987; Hutchinson and Fodor, 2012). To test the hypothesis that NUP85 might play a role in the interaction between PB1 and RanBP5, we co-transfected the HEK293T cells with plasmids expressing Flag-PB1, HA-RanBP5 and PA in the NUP85 siRNA or NUP85 expressing plasmids treated 293 T cells and detected the effect of NUP85 knockdown or overexpression on the PB1 and RanBP5 interaction by co-immunoprecipitation. The results showed that PB1 bound to RanBP5 as reported previously, but the relative amount of PB1 binding to RanBP5 was obviously decreased when NUP85 was knocked down and significantly increased when NUP85 was overexpressed (Figure 6A). The results suggest that NUP85 is involved in the interaction between PB1 and RanBP5. Since knockdown of NUP85 impedes nuclear import of PB2, and PB2 relies on the interaction with importin α1, α3, α5, and α7 to mediate its nuclear import (Mukaigawa and Nayak, 1991; Tarendeau et al., 2007; Gabriel et al., 2011), we hypothesized that NUP85 knockdown might affect the interaction between PB2 and importin α. To test the hypothesis, we treated HEK293T cells with NUP85 siRNA or NUP85 expressing plasmids for 24 h, then transfected with plasmids expressing Flag-PB2 and HA-importin α. The results of immunoprecipitation showed that PB2 bound to all four isoforms of importin α, and NUP85 knockdown obviously reduced the amounts of importin α1 and importin α7 immunoprecipitated with PB2 (Figures 6B,E), but not the amounts of immunoprecipitated importin α3 and importin α5 (Figures 6C,D), indicating that NUP85 knockdown interfered with the interaction between PB2 with both importin α1 and importin α7. Accordingly, NUP85 overexpression obviously increased the relative amounts of importin α1 and importin α7 immunoprecipitated with PB2 (Figures 6B,E), but not the relative amounts of immunoprecipitated importin α3 and importin α5 (Figures 6C,D). These results suggest that NUP85 facilitates the nuclear import of PB1 by enhancing the interaction of PB1 with RanBP5, and it facilitates the nuclear import of PB2 by enhancing the interaction of PB2 with importin α1 and importin α7.

### IAV infection decreases NUP85 expression

**Figure 7:**
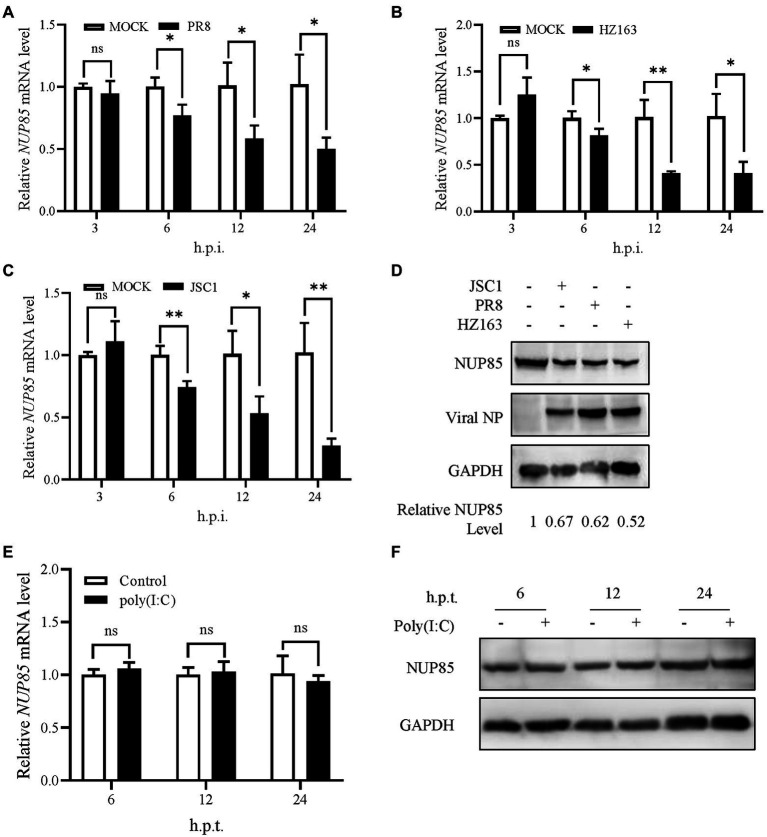
Influenza A virus infection decreased NUP85 expression. **(A–C)** A549 cells were infected with H1N1 PR8, H3N2 HZ163, and H9N2 JSC1, respectively (MOI = 0.1). Cells were harvested for RNA isolation at 3, 6, 12, and 24 h.p.i. The relative expression level of NUP85 was determined by qRT-PCR. Data are mean ± SD of three independent experiments. Significance is measured by two-way ANOVA test; * indicates *p* < 0.05, ** indicates *p* < 0.01. **(D)** A549 cells were infected with H9N2 JSC1, H1N1 PR8, and H3N2 HZ163 at indicated MOIs and harvested at 24 h.p.i. for Western blot analysis of NUP85, viral NP, and GAPDH protein levels. GAPDH was used as a loading control. **(E,F)** A549 cells were treated with 1 μg/ml poly(I:C) with (+) or without (−) transfection reagent for 6, 12, and 24 h, then collected for Western blot and qRT-PCR analysis of NUP85. Data are mean ± SD of three independent experiments. Significance is measured by unpaired *T*-test; * indicates *p* < 0.05. ** indicates *p* < 0.01.

To investigate NUP85 expression during IAV infection, we detected the mRNA and protein levels of NUP85 in A549 cells after infection with three strains of IAV (influenza A H1N1 PR8, H3N2 HZ163, H9N2 JSC1), respectively. The mRNA level of NUP85 was detected by qRT-PCR at different time points post-infection. We observed a gradual decrease in NUP85 mRNA level in the process of each virus infection, and the level decreased by half at 12 h.p.i. (Figures 7A–C). And about a two-fold decrease in NUP85 protein level was observed in the infected A549 cells at 24 h.p.i. (Figure 7D). Meanwhile, treatment with a synthetic analog of viral double-stranded RNA polyinosinic-polycytidylic acid (Poly(I: C)) does not decrease NUP85 expression (Figures 7E,F). These data demonstrated that NUP85 expression was downregulated upon IAV infection.

## Discussion

**Figure 8:**
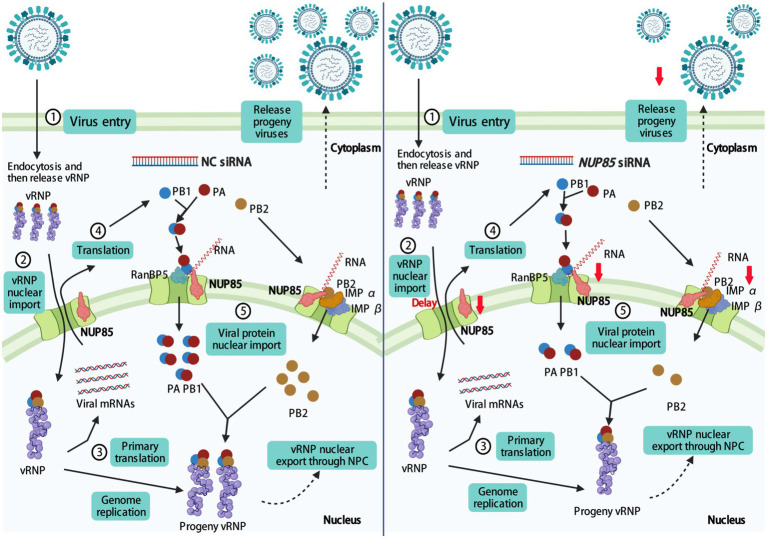
Proposed model for NUP85 facilitating nuclear import of vRNP and therefore promoting viral replication. Knockdown of NUP85 delays the nuclear import of vRNP, PB1, and PB2, which finally hinders viral replication. The figure was drawn at biorender.com under the agreement number of MT23YJ15J1.

Influenza A viruses are responsible for seasonal flu and even some pandemics. Several antivirals were developed for binding the viruses. Due to the rapid mutation of the viral genes, the evolution of resistance to the antiviral vaccine and therapy is rapid (Peacock et al., 2019). Understanding the interaction of virus and host factors would be of great importance in developing novel, effective antivirals. After influenza A virus invades the host cell, vRNPs are released into the cytoplasm, then they enter the nucleus through the nuclear pore for viral gene transcription and replication (Banerjee et al., 2014; Miyake et al., 2019; Sempere Borau and Stertz, 2021). IAV gene transcription and genome replication are mediated by the RNA-dependent RNA polymerase, which comprises PB1, PB2, and PA. PB1 is a critical factor of polymerase complex, and its N-terminal region binds to the C-terminal region of PA. The C-terminal region of PB1 interacts with the N-terminal region of PB2 (Te Velthuis and Fodor, 2016). In addition, NP interacts with PB1 and PB2 but not with PA (Biswas et al., 1998). For successful viral transcription and replication, vRNPs must pass through the NPC to the nucleus for primary gene transcription. Following the primary protein synthesis in the cytoplasm, newly formed viral proteins, including PB2, NP, PB1, and PA, are translocated back into the nucleus for vRNP assembly. Then the assembled progeny vRNP would be exported from the nuclear to the cytoplasm, and it finally reaches the cell membrane for progeny virus budding and release (Eisfeld et al., 2015; Peacock et al., 2019). In this study, we demonstrated that NUP85 interacted with the PB1 and PB2 in an RNA-dependent manner. Knockdown of NUP85 delayed the nuclear localization of vRNP in the early life cycle, weakened the binding of PB1 to RanBP5 and the binding of PB2 to importin α1 and importin α7, therefore leading to a smaller amount of nuclear PB1 and PB2, and further resulting in the reduced polymerase activity and suppressed virus replication. A proposed model of the mechanism NUP85 utilizes in the IAV infection is illustrated in Figure 8.

It has been well studied that vRNP utilizes the nuclear localization signals (NLSs) of NP for its nuclear translocation *via* the classic nuclear import pathway (Naito et al., 2007a; Hutchinson and Fodor, 2012). But whether NLSs of the polymerase subunits also contribute to the import of RNPs remains to be determined (Wu and Pante, 2009; Hutchinson and Fodor, 2012). In this study, we showed that NUP85 interacted with PB1 and PB2 in an RNA-dependent manner rather than with NP and PA. As RNA polymerase and nucleoprotein, all IAV RNP subunits are capable of binding viral RNA. NP can bind small RNA as well (Labaronne et al., 2016). What kind of RNA mediating interaction of NUP85 with PB1 and PB2 is not clear and worthy of further study. Knockdown of NUP85 significantly delayed vRNP entry into the nucleus at 3 h post-infection, inhibiting polymerase activity and suppressing viral replication. The study may provide a new possibility that NLSs of PB1 and PB2 also play a specific role in the nuclear import of RNPs. The classic IMP α-IMP β1 pathway used by viral protein includes cargo, importin-α, and importin-β1 (Tarendeau et al., 2007; Gabriel et al., 2008; Lange et al., 2010). The NLS-containing cargo protein binds to importin-α, and importin-α binds to importin-β1 through its N-terminal IBB domain, which constructs the complex of cargo-importin-α-importin-β1 (Cingolani et al., 1999; Lott et al., 2010). It has been shown that the ternary complex (cargo-importin-α-importin-β1) or binary complex (cargo-RanBP5) is transported through NPC by the interaction of importin-β with phenylalanine and glycine (FG)-riched nuclear pore proteins (Beck and Hurt, 2017; Hampoelz et al., 2019). Interestingly, NUP85 does not contain FG-repeat, which implies that nuclear transporter factors RanBP5 or importin-β1 may not bind to NUP85. Our study reveals that NUP85 silencing inhibits the nuclear entry of PB1 and PB2, suggesting that NUP85 is indeed associated with cargo-importin-α -Importin-β1 and cargo-RanBP5 complex. But the mechanism remains elusive, and it could be one of the future directions.

NUP85 is a part of subcomplex Nup107-160, which forms the out ring of NPC and is critical for NPC assembly (Walther et al., 2003). NUP85 is also a cytoplasmic protein (known as FROUNT), and its overexpression amplified the chemokine-elicited PI(3)K–Rac–lamellipodium protrusion cascade (Terashima et al., 2005). We tried to overexpress NUP85 to test its effect on vRNP import and viral replication. Still, we failed it as the cells became too unwell to do viral infection after NUP85 introduction either by transient transfection or by lentivirus transduction. It seems excessive NUP85 is not friendly to the A549 cells cell, and whether excessive NUP85 affected normal cellular metabolism due to NUP85 accumulating in the cytoplasm is elusive and worthy of further investigation. NUP85 interacts with HIV Tat protein in the cellular nucleus. Here, we showed that NUP85 interacted with IAV PB1 and PB2 protein, which was required for vRNP, PB1, and PB2 import into the nucleus. Other NUPs have also been shown to be essential for IAV infection. NUP62 is required for the nuclear export of IAV mRNA and vRNA (Morita et al., 2013). NUP93 is involved in the nuclear export of IAV RNA (Furusawa et al., 2018). NUP98 interacts with IAV NS2/nuclear export protein through its FG repeat, and overexpression of the domain inhibits virus propagation (Chen et al., 2010). In addition, the influenza A virus down-regulates Nup98 to facilitate its infection (Satterly et al., 2007). Moreover, Panda et al. showed that Nup98 directly binds to the promoters of virus-induced genes and promotes antiviral gene expression (Panda et al., 2014). But we did not see any difference in the mRNA levels of multiple immune-related factors in the NUP85 siRNA and NC siRNA-treated A549 cells after influenza A virus JSC1 H9N2 infection. It indicated knockdown of NUP85 did not affect the antiviral status of the infected cells. Therefore, the significantly decreased viral replication is mainly due to the decreased RNP import into the nucleus in the NUP85 knockdown cells. Furthermore, NUP85 protein expression significantly decreased during IAV infection (Figure 7). It is possible that the host cells used some strategies that merit further investigation to alter the NPC against IAV infection. NPC components would be the potential targets for influenza treatments.

## Conclusion

In conclusion, our data showed that NUP85 is a novel PB1 and PB2 binding protein and is pivotal for IAV replication. NUP85 knockdown impedes the nuclear import of vRNP, PB1 and PB2, thus inhibiting IAV replication. Importantly, we uncovered the underlying mechanisms that NUP85 assists the formation of a complex of viral PB2 and importin α1 and importin α7, PB1 and RanBP5 also. These results will advance our understanding that host proteins regulate the RNP nuclear import of IAV.

## Data availability statement

The original contributions presented in the study are included in the article/supplementary material, further inquiries can be directed to the corresponding author.

## Author contributions

YHL and YL designed the experiments, drafted the manuscript, and revised the manuscript. YHL, HW, MQH, and DW performed the experiments. YHL, YXH, KZ, and YL analyzed and interpreted the data. All authors contributed to the article and approved the submitted version.

## Funding

This work was supported by the National Natural Science Foundation of China (31872837), the National Program on Key Research Project of China (2019YFE0103900) as well as the European Union’s Horizon 2020 Research and Innovation Program under grant agreement no. 861917—SAFFI, the National Science Foundation for Distinguished Young Scholars (32102620), and the Natural Science Foundation of Zhejiang Province (LZ22C180004).

## Conflict of interest

The authors declare that the research was conducted in the absence of any commercial or financial relationships that could be construed as a potential conflict of interest.

## Publisher’s note

All claims expressed in this article are solely those of the authors and do not necessarily represent those of their affiliated organizations, or those of the publisher, the editors and the reviewers. Any product that may be evaluated in this article, or claim that may be made by its manufacturer, is not guaranteed or endorsed by the publisher.

## References

[ref1] Banerjee I. Miyake Y. Nobs S. P. Schneider C. Horvath P. Kopf M.. (2014). Influenza A virus uses the aggresome processing machinery for host cell entry. Science 346, 473–477. doi: 10.1126/science.1257037, PMID: 25342804

[ref2] Beck M. Hurt E. (2017). The nuclear pore complex: understanding its function through structural insight. Nat. Rev. Mol. Cell Biol. 18, 73–89. doi: 10.1038/nrm.2016.147, PMID: 27999437

[ref3] Biswas S. K. Boutz P. L. Nayak D. P. (1998). Influenza virus nucleoprotein interacts with influenza virus polymerase proteins. J. Virol. 72, 5493–5501. doi: 10.1128/JVI.72.7.5493-5501.1998, PMID: 9621005 PMC110190

[ref4] Brass A. L. Dykxhoorn D. M. Benita Y. Yan N. Engelman A. Xavier R. J.. (2008). Identification of host proteins required for HIV infection through a functional genomic screen. Science 319, 921–926. doi: 10.1126/science.1152725, PMID: 18187620

[ref5] Chen J. Huang S. Chen Z. (2010). Human cellular protein nucleoporin hNup98 interacts with influenza A virus NS2/nuclear export protein and overexpression of its GLFG repeat domain can inhibit virus propagation. J. Gen. Virol. 91, 2474–2484. doi: 10.1099/vir.0.022681-0, PMID: 20554795

[ref6] Cingolani G. Petosa C. Weis K. Muller C. W. (1999). Structure of importin-beta bound to the IBB domain of importin-alpha. Nature 399, 221–229. doi: 10.1038/20367, PMID: 10353244

[ref7] Deng T. Engelhardt O. G. Thomas B. Akoulitchev A. V. Brownlee G. G. Fodor E. (2006). Role of ran binding protein 5 in nuclear import and assembly of the influenza virus RNA polymerase complex. J. Virol. 80, 11911–11919. doi: 10.1128/JVI.01565-06, PMID: 17005651 PMC1676300

[ref8] Eisfeld A. J. Neumann G. Kawaoka Y. (2015). At the Centre: influenza A virus ribonucleoproteins. Nat. Rev. Microbiol. 13, 28–41. doi: 10.1038/nrmicro3367, PMID: 25417656 PMC5619696

[ref9] Furusawa Y. Yamada S. Kawaoka Y. (2018). Host factor Nucleoporin 93 is involved in the nuclear export of influenza virus RNA. Front. Microbiol. 9:1675. doi: 10.3389/fmicb.2018.01675, PMID: 30087672 PMC6066526

[ref10] Gabriel G. Herwig A. Klenk H. D. (2008). Interaction of polymerase subunit PB2 and NP with importin alpha1 is a determinant of host range of influenza A virus. PLoS Pathog. 4:e11. doi: 10.1371/journal.ppat.0040011, PMID: 18248089 PMC2222953

[ref11] Gabriel G. Klingel K. Otte A. Thiele S. Hudjetz B. Arman-Kalcek G.. (2011). Differential use of importin-alpha isoforms governs cell tropism and host adaptation of influenza virus. Nat. Commun. 2:156. doi: 10.1038/ncomms1158, PMID: 21245837 PMC3105303

[ref12] Gao Q. Yang C. Ren C. Zhang S. Gao X. Jin M.. (2020). Eukaryotic translation elongation factor 1 Delta inhibits the nuclear import of the nucleoprotein and PA-PB1 heterodimer of influenza A virus. J. Virol. 95:01391-20. doi: 10.1128/JVI.01391-20, PMID: 33087462 PMC7944447

[ref13] Gautier V. W. Gu L. O'Donoghue N. Pennington S. Sheehy N. Hall W. W. (2009). In vitro nuclear interactome of the HIV-1 tat protein. Retrovirology 6:47. doi: 10.1186/1742-4690-6-47, PMID: 19454010 PMC2702331

[ref14] Gorlich D. Vogel F. Mills A. D. Hartmann E. Laskey R. A. (1995). Distinct functions for the two importin subunits in nuclear protein import. Nature 377, 246–248. doi: 10.1038/377246a0, PMID: 7675110

[ref15] Hampoelz B. Andres-Pons A. Kastritis P. Beck M. (2019). Structure and assembly of the nuclear pore complex. Annu. Rev. Biophys. 48, 515–536. doi: 10.1146/annurev-biophys-052118-115308 30943044

[ref16] Harel A. Orjalo A. V. Vincent T. Lachish-Zalait A. Vasu S. Shah S.. (2003). Removal of a single pore subcomplex results in vertebrate nuclei devoid of nuclear pores. Mol. Cell 11, 853–864. doi: 10.1016/s1097-2765(03)00116-3, PMID: 12718872

[ref17] Huet S. Avilov S. V. Ferbitz L. Daigle N. Cusack S. Ellenberg J. (2010). Nuclear import and assembly of influenza A virus RNA polymerase studied in live cells by fluorescence cross-correlation spectroscopy. J. Virol. 84, 1254–1264. doi: 10.1128/JVI.01533-09, PMID: 19906916 PMC2812328

[ref18] Hutchinson E. C. Fodor E. (2012). Nuclear import of the influenza A virus transcriptional machinery. Vaccine 30, 7353–7358. doi: 10.1016/j.vaccine.2012.04.085, PMID: 22652398

[ref19] Hutchinson E. C. Orr O. E. Man Liu S. Engelhardt O. G. Fodor E. (2011). Characterization of the interaction between the influenza A virus polymerase subunit PB1 and the host nuclear import factor ran-binding protein 5. J. Gen. Virol. 92, 1859–1869. doi: 10.1099/vir.0.032813-0, PMID: 21562121

[ref20] Jones I. M. Reay P. A. Philpott K. L. (1986). Nuclear location of all three influenza polymerase proteins and a nuclear signal in polymerase PB2. EMBO J. 5, 2371–2376. doi: 10.1002/j.1460-2075.1986.tb04506.x, PMID: 3023071 PMC1167122

[ref21] Kawaguchi A. Momose F. Nagata K. (2011). Replication-coupled and host factor-mediated encapsidation of the influenza virus genome by viral nucleoprotein. J. Virol. 85, 6197–6204. doi: 10.1128/JVI.00277-11, PMID: 21507964 PMC3126543

[ref22] Labaronne A. Swale C. Monod A. Schoehn G. Crepin T. Ruigrok R. W. (2016). Binding of RNA by the nucleoproteins of influenza viruses A and B. Viruses 8:247. doi: 10.3390/v8090247, PMID: 27649229 PMC5035961

[ref23] Lange A. McLane L. M. Mills R. E. Devine S. E. Corbett A. H. (2010). Expanding the definition of the classical bipartite nuclear localization signal. Traffic 11, 311–323. doi: 10.1111/j.1600-0854.2009.01028.x, PMID: 20028483 PMC2886731

[ref24] Li B. Clohisey S. M. Chia B. S. Wang B. Cui A. Eisenhaure T.. (2020). Genome-wide CRISPR screen identifies host dependency factors for influenza A virus infection. Nat. Commun. 11:164. doi: 10.1038/s41467-019-13965-x, PMID: 31919360 PMC6952391

[ref25] Lim R. Y. Ullman K. S. Fahrenkrog B. (2008). Biology and biophysics of the nuclear pore complex and its components. Int. Rev. Cell Mol. Biol. 267, 299–342. doi: 10.1016/S1937-6448(08)00632-1, PMID: 18544502 PMC4366138

[ref26] Lin D. H. Hoelz A. (2019). The structure of the nuclear pore complex (an update). Annu. Rev. Biochem. 88, 725–783. doi: 10.1146/annurev-biochem-062917-011901, PMID: 30883195 PMC6588426

[ref27] Lott K. Bhardwaj A. Mitrousis G. Pante N. Cingolani G. (2010). The importin beta binding domain modulates the avidity of importin beta for the nuclear pore complex. J. Biol. Chem. 285, 13769–13780. doi: 10.1074/jbc.M109.095760, PMID: 20197273 PMC2859540

[ref28] Luo W. Zhang J. Liang L. Wang G. Li Q. Zhu P.. (2018). Phospholipid scramblase 1 interacts with influenza A virus NP, impairing its nuclear import and thereby suppressing virus replication. PLoS Pathog. 14:e1006851. doi: 10.1371/journal.ppat.1006851, PMID: 29352288 PMC5792031

[ref29] Lutz A. Dyall J. Olivo P. D. Pekosz A. (2005). Virus-inducible reporter genes as a tool for detecting and quantifying influenza A virus replication. J. Virol. Methods 126, 13–20. doi: 10.1016/j.jviromet.2005.01.016, PMID: 15847914 PMC1698269

[ref30] Matsuoka Y. Matsumae H. Katoh M. Eisfeld A. J. Neumann G. Hase T.. (2013). A comprehensive map of the influenza A virus replication cycle. BMC Syst. Biol. 7:97. doi: 10.1186/1752-0509-7-97, PMID: 24088197 PMC3819658

[ref31] Miyake Y. Keusch J. J. Decamps L. Ho-Xuan H. Iketani S. Gut H.. (2019). Influenza virus uses transportin 1 for vRNP debundling during cell entry. Nat. Microbiol. 4, 578–586. doi: 10.1038/s41564-018-0332-2, PMID: 30692667

[ref32] Moeller A. Kirchdoerfer R. N. Potter C. S. Carragher B. Wilson I. A. (2012). Organization of the influenza virus replication machinery. Science 338, 1631–1634. doi: 10.1126/science.1227270, PMID: 23180774 PMC3578580

[ref33] Morita M. Kuba K. Ichikawa A. Nakayama M. Katahira J. Iwamoto R.. (2013). The lipid mediator protectin D1 inhibits influenza virus replication and improves severe influenza. Cell 153, 112–125. doi: 10.1016/j.cell.2013.02.027, PMID: 23477864

[ref34] Muhlbauer D. Dzieciolowski J. Hardt M. Hocke A. Schierhorn K. L. Mostafa A.. (2015). Influenza virus-induced caspase-dependent enlargement of nuclear pores promotes nuclear export of viral ribonucleoprotein complexes. J. Virol. 89, 6009–6021. doi: 10.1128/JVI.03531-14, PMID: 25810542 PMC4442457

[ref35] Mukaigawa J. Nayak D. P. (1991). Two signals mediate nuclear localization of influenza virus (A/WSN/33) polymerase basic protein 2. J. Virol. 65, 245–253. doi: 10.1128/JVI.65.1.245-253.1991, PMID: 1985200 PMC240511

[ref36] Naito T. Kiyasu Y. Sugiyama K. Kimura A. Nakano R. Matsukage A.. (2007a). An influenza virus replicon system in yeast identified tat-SF1 as a stimulatory host factor for viral RNA synthesis. Proc. Natl. Acad. Sci. U. S. A. 104, 18235–18240. doi: 10.1073/pnas.0705856104, PMID: 17991777 PMC2084326

[ref37] Naito T. Momose F. Kawaguchi A. Nagata K. (2007b). Involvement of Hsp90 in assembly and nuclear import of influenza virus RNA polymerase subunits. J. Virol. 81, 1339–1349. doi: 10.1128/JVI.01917-06, PMID: 17121807 PMC1797515

[ref38] Neumann G. Noda T. Kawaoka Y. (2009). Emergence and pandemic potential of swine-origin H1N1 influenza virus. Nature 459, 931–939. doi: 10.1038/nature08157, PMID: 19525932 PMC2873852

[ref39] Noda T. Kawaoka Y. (2010). Structure of influenza virus ribonucleoprotein complexes and their packaging into virions. Rev. Med. Virol. 20, 380–391. doi: 10.1002/rmv.666, PMID: 20853340 PMC6029254

[ref40] O'Neill R. E. Jaskunas R. Blobel G. Palese P. Moroianu J. (1995). Nuclear import of influenza virus RNA can be mediated by viral nucleoprotein and transport factors required for protein import. J. Biol. Chem. 270, 22701–22704. doi: 10.1074/jbc.270.39.22701, PMID: 7559393

[ref41] Panda D. Pascual-Garcia P. Dunagin M. Tudor M. Hopkins K. C. Xu J.. (2014). Nup98 promotes antiviral gene expression to restrict RNA viral infection in drosophila. Proc. Natl. Acad. Sci. U. S. A. 111, E3890–E3899. doi: 10.1073/pnas.1410087111, PMID: 25197089 PMC4169978

[ref42] Peacock T. P. Sheppard C. M. Staller E. Barclay W. S. (2019). Host determinants of influenza RNA synthesis. Annu. Rev. Virol. 6, 215–233. doi: 10.1146/annurev-virology-092917-043339, PMID: 31283439

[ref43] Resa-Infante P. Jorba N. Zamarreno N. Fernandez Y. Juarez S. Ortin J. (2008). The host-dependent interaction of alpha-importins with influenza PB2 polymerase subunit is required for virus RNA replication. PLoS One 3:e3904. doi: 10.1371/journal.pone.0003904, PMID: 19066626 PMC2588535

[ref44] Resa-Infante P. Recuero-Checa M. A. Zamarreno N. Llorca O. Ortin J. (2010). Structural and functional characterization of an influenza virus RNA polymerase-genomic RNA complex. J. Virol. 84, 10477–10487. doi: 10.1128/JVI.01115-10, PMID: 20702645 PMC2950564

[ref45] Satterly N. Tsai P. L. van Deursen J. Nussenzveig D. R. Wang Y. Faria P. A.. (2007). Influenza virus targets the mRNA export machinery and the nuclear pore complex. Proc. Natl. Acad. Sci. U. S. A. 104, 1853–1858. doi: 10.1073/pnas.0610977104, PMID: 17267598 PMC1794296

[ref46] Sempere Borau M. Stertz S. (2021). Entry of influenza A virus into host cells - recent progress and remaining challenges. Curr. Opin. Virol. 48, 23–29. doi: 10.1016/j.coviro.2021.03.001, PMID: 33838498

[ref47] Senbas Akyazi B. Pirincal A. Kawaguchi A. Nagata K. Turan K. (2020). Interaction of influenza A virus NS2/NEP protein with the amino-terminal part of Nup214. Turk. J. Biol. 44, 82–92. doi: 10.3906/biy-1909-49, PMID: 32256144 PMC7129063

[ref48] Smith G. L. Levin J. Z. Palese P. Moss B. (1987). Synthesis and cellular location of the ten influenza polypeptides individually expressed by recombinant vaccinia viruses. Virology 160, 336–345. doi: 10.1016/0042-6822(87)90004-3, PMID: 3310381

[ref49] Tarendeau F. Boudet J. Guilligay D. Mas P. J. Bougault C. M. Boulo S.. (2007). Structure and nuclear import function of the C-terminal domain of influenza virus polymerase PB2 subunit. Nat. Struct. Mol. Biol. 14, 229–233. doi: 10.1038/nsmb1212, PMID: 17310249

[ref50] Taubenberger J. K. Kash J. C. Morens D. M. (2019). The 1918 influenza pandemic: 100 years of questions answered and unanswered. Sci. Transl. Med. 11:eaau5485. doi: 10.1126/scitranslmed.aau5485, PMID: 31341062 PMC11000447

[ref51] Taubenberger J. K. Morens D. M. (2008). The pathology of influenza virus infections. Annu. Rev. Pathol. 3, 499–522. doi: 10.1146/annurev.pathmechdis.3.121806.154316, PMID: 18039138 PMC2504709

[ref52] Te Velthuis A. J. Fodor E. (2016). Influenza virus RNA polymerase: insights into the mechanisms of viral RNA synthesis. Nat. Rev. Microbiol. 14, 479–493. doi: 10.1038/nrmicro.2016.87, PMID: 27396566 PMC4966622

[ref53] Terashima Y. Onai N. Murai M. Enomoto M. Poonpiriya V. Hamada T.. (2005). Pivotal function for cytoplasmic protein FROUNT in CCR2-mediated monocyte chemotaxis. Nat. Immunol. 6, 827–835. doi: 10.1038/ni1222, PMID: 15995708

[ref54] Terashima Y. Toda E. Itakura M. Otsuji M. Yoshinaga S. Okumura K.. (2020). Targeting FROUNT with disulfiram suppresses macrophage accumulation and its tumor-promoting properties. Nat. Commun. 11:609. doi: 10.1038/s41467-020-14338-5, PMID: 32001710 PMC6992764

[ref55] Terry L. J. Wente S. R. (2007). Nuclear mRNA export requires specific FG nucleoporins for translocation through the nuclear pore complex. J. Cell Biol. 178, 1121–1132. doi: 10.1083/jcb.200704174, PMID: 17875746 PMC2064648

[ref56] Toda E. Terashima Y. Esaki K. Yoshinaga S. Sugihara M. Kofuku Y.. (2014). Identification of a binding element for the cytoplasmic regulator FROUNT in the membrane-proximal C-terminal region of chemokine receptors CCR2 and CCR5. Biochem. J. 457, 313–322. doi: 10.1042/BJ20130827, PMID: 24128342

[ref57] Toda E. Terashima Y. Sato T. Hirose K. Kanegasaki S. Matsushima K. (2009). FROUNT is a common regulator of CCR2 and CCR5 signaling to control directional migration. J. Immunol. 183, 6387–6394. doi: 10.4049/jimmunol.0803469, PMID: 19841162

[ref58] Walther T. C. Alves A. Pickersgill H. Loiodice I. Hetzer M. Galy V.. (2003). The conserved Nup107-160 complex is critical for nuclear pore complex assembly. Cell 113, 195–206. doi: 10.1016/s0092-8674(03)00235-6, PMID: 12705868

[ref59] Wu W. W. Pante N. (2009). The directionality of the nuclear transport of the influenza A genome is driven by selective exposure of nuclear localization sequences on nucleoprotein. Virol. J. 6:68. doi: 10.1186/1743-422X-6-68, PMID: 19490630 PMC2694790

[ref60] Wu W. W. Sun Y. H. Pante N. (2007). Nuclear import of influenza A viral ribonucleoprotein complexes is mediated by two nuclear localization sequences on viral nucleoprotein. Virol. J. 4:49. doi: 10.1186/1743-422X-4-49, PMID: 17547769 PMC1891284

[ref61] Yang C. Liu X. Gao Q. Cheng T. Xiao R. Ming F.. (2018). The Nucleolar protein LYAR facilitates Ribonucleoprotein assembly of influenza A virus. J. Virol. 92:01042-18. doi: 10.1128/JVI.01042-18, PMID: 30209172 PMC6232469

[ref62] Zannettino A. C. Psaltis P. J. Gronthos S. (2008). Home is where the heart is: via the FROUNT. Cell Stem Cell 2, 513–514. doi: 10.1016/j.stem.2008.05.012, PMID: 18522840

[ref63] Zhou B. Li Y. Halpin R. Hine E. Spiro D. J. Wentworth D. E. (2011). PB2 residue 158 is a pathogenic determinant of pandemic H1N1 and H5 influenza a viruses in mice. J. Virol. 85, 357–365. doi: 10.1128/JVI.01694-10, PMID: 20962098 PMC3014153

